# Deposition of Very-Low-Hydrogen-Containing Silicon at a Low Temperature Using Very-High-Frequency (162 MHz) SiH_4_ Plasma

**DOI:** 10.3390/mi13020173

**Published:** 2022-01-24

**Authors:** Ki Seok Kim, You-Jin Ji, Ki-Hyun Kim, Ji-Eun Kang, Albert Rogers Ellingboe, Geun Young Yeom

**Affiliations:** 1 Research Laboratory of Electronics, Massachusetts Institute of Technology, Cambridge, MA 02139, USA; kiseok@mit.edu; 2 School of Advanced Materials Science and Engineering, Sungkyunkwan University, 2066 Seobu-ro, Jangan-gu, Suwon-si 16419, Korea; gotnr3205@skku.edu (Y.-J.J.); ngdu@skku.edu (K.-H.K.); wldms8999@skku.edu (J.-E.K.); 3 Plasma Research Laboratory, School of Physical Sciences and NCPST, Dublin City University, D09 V209 Dublin, Ireland; albert.ellingboe@dcu.ie; 4 SKKU Advanced Institute of Nano Technology (SAINT), Sungkyunkwan University, 2066 Seobu-ro, Jangan-gu, Suwon-si 16419, Korea

**Keywords:** very high frequency (VHF), multi-split electrode, low-temperature polysilicon (LTPS)

## Abstract

Low-hydrogen-containing amorphous silicon (a-Si) was deposited at a low temperature of 80 °C using a very high frequency (VHF at 162 MHz) plasma system with multi-split electrodes. Using the 162 MHz VHF plasma system, a high deposition rate of a-Si with a relatively high deposition uniformity of 6.7% could be obtained due to the formation of high-ion-density (>10^11^ cm^−3^) plasma with SiH_4_ and a lack of standing waves by using small multi-split electrodes. The increase in the radio frequency (RF) power decreased the hydrogen content in the deposited silicon film and, at a high RF power of 2000 W, a-Si with a low hydrogen content of 3.78% could be deposited without the need for a dehydrogenation process. The crystallization of the a-Si by ultraviolet (UV) irradiation showed that the a-Si can be crystallized with a crystallinity of 0.8 and a UV energy of 80 J without dehydrogenation. High-resolution transmission electron microscopy showed that the a-Si deposited by the VHF plasma was a very small nanocrystalline-like a-Si and the crystalline size significantly grew with the UV irradiation. We believe that the VHF (162 MHz) multi-split plasma system can be used for a low-cost low-temperature polysilicon (LTPS) process.

## 1. Introduction

Among thin-film transistors (TFTs), low-temperature polysilicon (LTPS) TFTs have significant advantages in device characteristics, such as a low threshold voltage, steep subthreshold swing, high voltage/current reliability, and high yield [1]. They are widely applied in high-resolution liquid crystal displays (LCDs), image sensors, photovoltaics, active-matrix organic light emitting diodes (AMOLEDs), and to flexible displays, because their mobility is about 100 times faster than that of a-Si thin-film transistors (TFTs) [2,3].

The process steps used for fabricating LTPS TFTs consist of (1st step) a-Si deposition; (2nd step) dehydrogenation; and (3rd step) crystallization using various methods, such as rapid thermal annealing (RTA) [4], metal-induced lateral crystallization (MILC) [5,6], plasma surface treatment [7,8], and lamp heating [9,10,11,12], as well as excimer laser annealing (ELA) [2,13,14,15]. Among these, a-Si is generally deposited by plasma-enhanced chemical vapor deposition (PECVD). The a-Si deposited by PECVD generally contains hydrogen at ~10% or more [2]. When the crystallization process is carried out using high hydrogen containing a-Si for polysilicon formation, hydrogen diffuses quickly, which makes the surface rough and porous [16]. Therefore, a dehydrogenation process is required and the process is performed during a lengthy heat treatment at ~450 °C before the crystallization process, which results in high equipment investment costs.

In this study, for the simplification of the LTPS process requiring no dehydration, a-Si was deposited at a low temperature of 80 °C using a very high frequency (VHF, 162 MHz) plasma system with multi-split electrodes. By using a VHF (162 MHz) plasma system, it is possible to form a high-quality thin film by increasing the gas dissociation rate compared to that of conventional 13.56 MHz plasma. The advantages of the use of VHF plasma compared to conventional 13.56 MHz in thin-film deposition and surface treatment have been clearly demonstrated by our previous studies [17,18,19], and the reasons for this are the low electron temperature and high vibrational temperature of VHF (162 MHz) plasma [19]. Since the VHF plasma generated with multi-split electrodes can sufficiently decompose the silane (SiH_4_) gas without showing a standing wave effect [20,21,22] and, therefore, can deposit a-Si with a very low hydrogen content uniformly, highly crystallized polysilicon could be fabricated through a crystallization process using an ultraviolet (UV) lamp without a dehydrogenation process.

## 2. Materials and Methods

**Figure 1:**
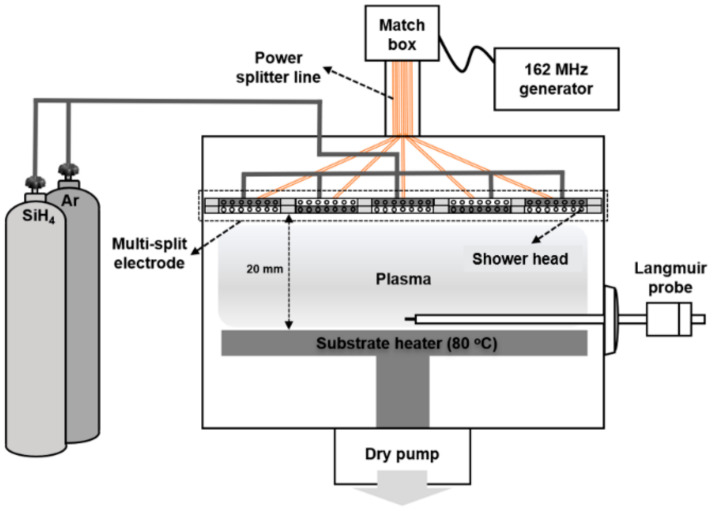
Schematic diagram of the VHF (162 MHz) plasma system with multi-split electrodes.

**Figure 2:**
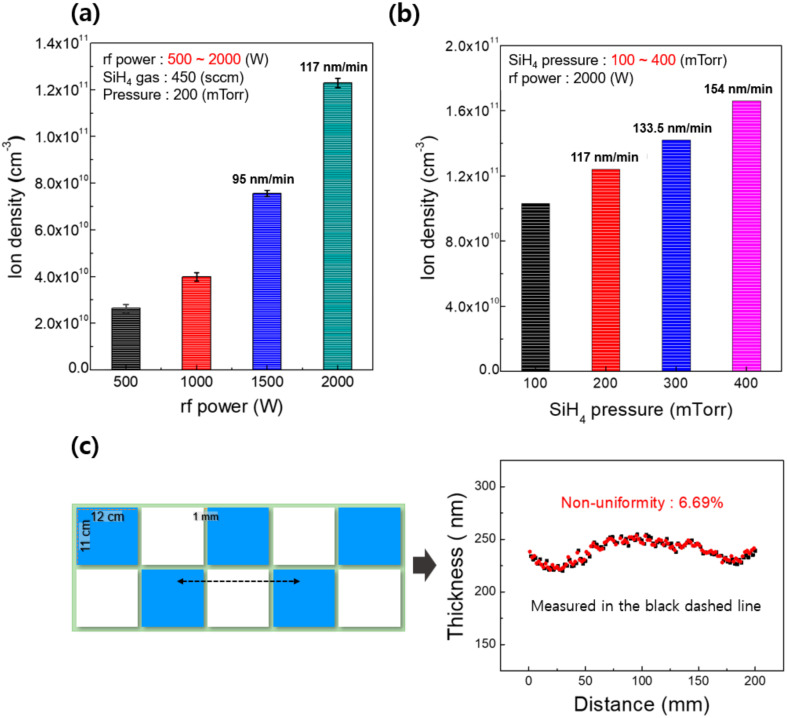
Ion density measured using a Langmuir probe (**a**) at different RF powers (500~2000 W) and (**b**) different SiH_4_ pressures (100~400 mTorr) in a VHF (162 MHz) plasma source with multi-split electrodes. (**c**) The schematic diagram of multi-split electrodes, with the thickness/non-uniformity measured at 1 mm intervals in a 200 mm region of an a-Si thin film deposited using the VHF (162 MHz) plasma source at 2000 W, 200 mTorr, and 80 °C substrate temperature.

Figure 1 is a schematic diagram of the VHF (162 MHz) plasma system with multi-split electrodes used in this experiment. The 162 MHz RF power was equally supplied to each multi-split electrode through the matcher and a power splitter system. A detailed description of the VHF (162 MHz) plasma system with multi-split electrodes can be found elsewhere [15,16,22,23,24,25]. Each electrode was made of a rectangular tile-shaped (11 cm × 12 cm) anodized aluminum with holes for the showerhead to distribute SiH_4_ gas uniformly in the process chamber. The electrodes were located ~1 cm apart regularly in pairs, and the split RF power was applied to two adjacent tile pair electrodes (shown as blue and white tiles in Figure 2).

The a-Si was deposited on Corning glass substrates at the operating pressure of 200 mTorr SiH_4_ and a low temperature of 80 °C for 2 min while the 162 MHz RF power was varied from 500 to 2000 W. The deposited a-Si films were crystallized by ultraviolet (UV) irradiation using a UV lamp with a wavelength of 254 nm. The energy of the UV lamp was varied from 50 to 80 J.

The surface morphology of the deposited a-Si was observed by field emission scanning electron microscopy (FE-SEM, S4700, Hitachi, Tokyo, Japan). Secondary ion mass spectroscopy (D-SIMS, IMS 4FE7, Cameca, Gennevilliers, France) was carried out to extract the hydrogen contents in the deposited a-Si films using Cs^+^ ions with an impact energy of 14.5 kV. X-ray photoelectron spectroscopy (XPS, MultiLab 2000, Thermo VG, Waltham, MA USA, Mg Kα source) was performed to evaluate the chemical properties of deposited a-Si. The crystallinity of the UV-irradiated a-Si was measured by Raman spectroscopy (α 300 M +, WITEC, Eglfinger weg2, Germany) and transmittance electron microscopy (TEM, JEM-2100F, JEOL, Tokyo, Japan). A Langmuir probe (ALP-150, Impedans, Dublin, Ireland) was used to measure the ion density in the SiH_4_ plasmas.

## 3. Results and Discussion

Figure 2a,b show the ion density of SiH_4_ plasmas measured as a function of RF power at a fixed SiH_4_ pressure of 200 mTorr and as a function of SiH_4_ pressure at a fixed RF power of 2000 W, respectively. The ion density was measured using a Langmuir probe at the center of the process chamber near the substrate location. As shown in Figure 2a, the ion density was gradually increased by increasing the RF power from 500 to 2000, and a high plasma density of ≥10^11^ cm^−3^ could be obtained at 2000 W. The growth rates of a-Si at 1500 and 2000 W with a fixed SiH_4_ pressure of 200 mTorr were measured as 95 and 117 nm/min, respectively. Additionally, as shown in Figure 2b, as the SiH_4_ pressure was increased from 100 to 400 mTorr at a fixed RF power of 2000 W, the ion density was increased and a high ion density (> 10^11^ cm^−3^) was obtained at a pressure over 200 mTorr. On the other hand, conventional 13.56 MHz CCP showed a low plasma density at around 10^10^ cm^−3^ [26]. As the pressure increased from 200 to 400 mTorr, the growth rate also increased from 117 to 154 nm/min.

Figure 2c shows the thickness uniformity of a-Si film deposited at a fixed condition of 2000 W of RF power and 200 mTorr of SiH_4_. The deposition process was performed at the substrate temperature of 80 °C for 2 min using the rectangular multi-split electrodes shown in Figure 2c. The thickness of the deposited a-Si was measured along the black dash line (200 mm) with 1 mm intervals to evaluate the thickness uniformity across the electrode. The points of measurement include the positions of repeating electrode tile faces/boundaries. As shown in Figure 2c, the measured thickness non-uniformity was 6.69% and the average thickness of a-Si was 234 nm. In general, when a VHF is used, due to the short wavelength of the VHF compared to the size of the electrode, a standing wave effect causing non-uniformity along the electrode centerline can be observed. By using the multi-split electrodes with a size much smaller than the wavelength of the VHF, the standing wave effect was suppressed and resulting film was relatively uniform. Moreover, a high deposition rate of 117 nm/min was achieved due to the high dissociation rate of SiH_4_, even at a low deposition temperature. In our previous studies, we have demonstrated that 162 MHz plasma has significantly higher dissociation rates than 60 and 13.56 MHz plasmas, which can minimize hydrogen bonding in the thin film while improving the deposition rate [15,16].

**Figure 3:**
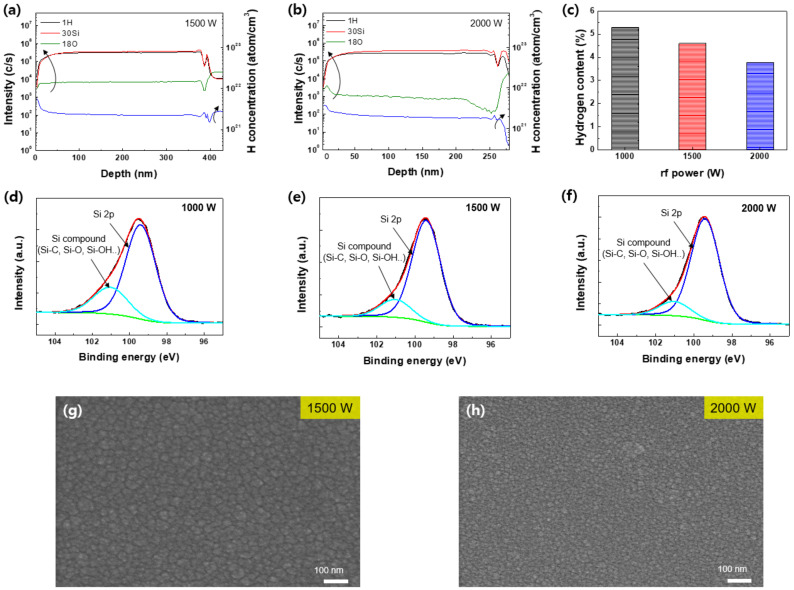
SIMS depth profile of a-Si films with different RF powers of (**a**) 1500 W and (**b**) 2000 W. (**c**) Hydrogen contents and (**d**–**f**) XPS Si 2p spectra of deposited a-Si at different RF powers (1000~2000 W). SEM images of a-Si deposited at (**g**) 1500 W and (**h**) 2000 W.

To investigate the influence of RF power on the hydrogen contents of a-Si, a-Si was deposited with different RF powers of 1000~2000 W. Figure 3a,b show SIMS data (left) on H, O, and Si for a-Si deposited with the RF powers of 1500 and 2000 W and the hydrogen concentration in the deposited a-Si calculated from the SIMS results (right). Using the relation of C_H_ = (N_H_/N_si_) × 100, where N_H_ is from SIMS results in Figure 3b and N_Si_ is 5 × 10^22^/cm^3^ from the literature [27,28], the hydrogen contents in the deposited a-Si films were calculated and the results are shown in Figure 3c. As shown in Figure 3c, the calculated hydrogen content in the a-Si film was decreased from 5.3 to 3.78% with the increasing RF power, which shows that the RF power can influence the hydrogen content in the films. During the deposition process, hydrogen atoms weakly bonded with Si atoms can be easily eliminated by ion bombardment. High RF power not only increases the dissociation rate of SiH_4_ but also increases the ion densities as shown in Figure 2a, causing more ion bombardment on the depositing a-Si surface in addition to increased atomic hydrogen flux [27,28,29,30]. Therefore, at the higher RF power conditions, H atoms adsorbed on the a-Si surface could be easily desorbed from a-Si surface, because the rate of H desorption is influenced by ion bombardment [31,32,33]. Figure 3d–f show the XPS narrow scan data of Si 2p of a-Si deposited with RF powers of 1000~2000 W. The XPS narrow scan data of the Si 2p were deconvoluted into Si 2p and Si compounds, which are composed of Si-C, Si-O, and Si-OH near the binding energy of 101~104 eV [34]. In the VHF (162 MHz) multi-split plasma system, the increase in RF power increased the dissociation rate of the SiH_4_ and decreased the content of Si compounds, indicating the growth of high-quality a-Si films at higher RF powers. Figure 3g,h show the top-view SEM images of a-Si corresponding to Figure 3a,b), respectively. Clearly, more nanocrystalline-like a-Si was observed at a higher RF power.

**Figure 4:**
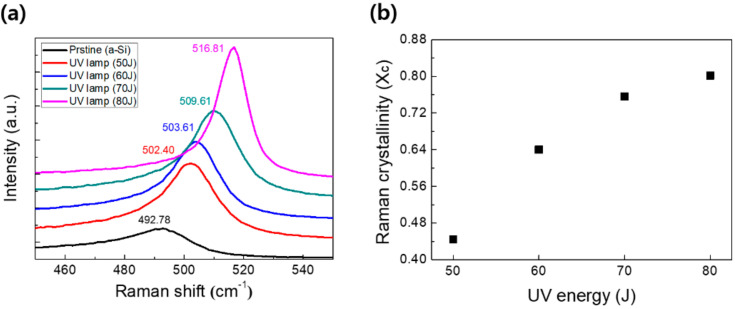
(**a**) Raman spectra and (**b**) Raman crystallinity (X_c_) of a-Si after the crystallization with different UV energies (50~80 J).

Based on the results obtained for the a-Si with a lower hydrogen content deposited at the higher RF power, the UV crystallization process was performed on the deposited a-Si film under the condition of an RF power of 2000 W, SiH_4_ flow rate of 450 sccm, working pressure of 200 mTorr, and substrate temperature of 80 °C. For the crystallization of the low-hydrogen-containing a-Si, a UV lamp with a wavelength of 254 nm was used to crystallize a-Si and the UV lamp was exposed for 1 min at a distance of 1 cm from the a-Si. Figure 4a shows the Raman spectra of the a-Si films before and after irradiation at different UV lamp energies to confirm the crystalline property of the irradiated a-Si. Untreated pristine a-Si shows a broad and low intensity of peaks centered at 492.78 cm^−1^. In contrast, the a-Si irradiated by the UV lamp with the energy of 50~80 J showed a relatively sharp peak blue-shifted with respect to the pristine a-Si, and the peak positions were closer to the crystalline Si Raman peak of 520 cm^−1^ at increasing UV energies. At the UV energy of 80 J, a sharp peak at 516.81 cm^−1^ was observed, indicating the high crystallinity of UV irradiated a-Si. Raman crystallinity can be estimated from the Raman shift of the UV irradiated a-Si by applying Equation (1) [35,36,37,38]:

X_c_ = (I_500_ + I_520_)/(aI_480_ + I_500_ + I_520_) (1)

where I_480_, I_500_, and I_520_ correspond to the amorphous, intermediate, and crystalline components, respectively. From the above equation, the crystallinities of a-Si irradiated with different UV energies are plotted in Figure 4b. As shown in Figure 4b, the Raman crystallinity was gradually improved from 0.45 to 0.8 as the UV energy was increased from 50 to 80 J.

**Figure 5:**
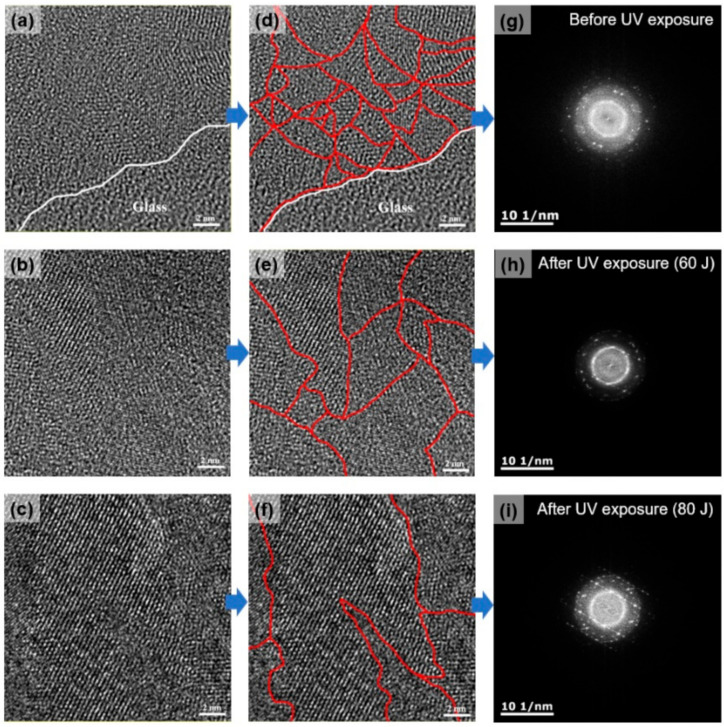
(**a**–**c**) HR-TEM image, (**d**–**f**) grain boundary indicated (red line) in the HR-TEM image, and (**g**–**i**) fast Fourier transform (FFT) pattern of as-deposited a-Si irradiated by UV lamp at 60 and 80 J.

An HR-TEM analysis was performed to confirm the morphology of the silicon thin films crystallized after the irradiation using the UV lamp. Figure 5 shows the HR-TEM images and FFT patterns of the a-Si films before and after the crystallization by the UV lamp (60 and 80 J). Figure 5a shows the small nanocrystalline-like grains of the a-Si before the UV exposure deposited using 2000 W of VHF (162 MHz) SiH_4_ plasma. In order to more easily distinguish the grain size, noticeable grain boundaries are shown as a red line in Figure 5d. Figure 5g is the FFT pattern measured for Figure 5d, and an amorphous ring pattern was confirmed due to the nanocrystalline-like grains. On the other hand, as shown in the HR-TEM images of Figure 5e,f, the grain size was gradually increased as the UV lamp energy was increased to 60 and 80 J, respectively, and strong polycrystalline phases were also observed in the FFT patterns, as shown in Figure 5h,i. These results demonstrate that low-hydrogen-containing a-Si deposited using a VHF (162 MHz) multi-split plasma source can be crystallized without a dehydrogenation process.

## 4. Conclusions

VHF (162 MHz) SiH_4_ plasmas with multi-split electrodes were investigated regarding the deposition of low-hydrogen-containing a-Si required for the crystallization of silicon without the use of a dehydrogenation process at a low substrate temperature of 80 °C uniformly without showing the standing wave effect. Increasing the VHF RF power to the SiH_4_ plasma not only increased the hydrogen dissociation but also increased the ion density in the plasma. By increasing the RF power in the deposited a-Si film, the hydrogen percentage was decreased to 3.78% at 2000 W of RF power, while a decrease in the impurities in the film, such as carbon/oxygen, was also achieved. The low content of impurities such as hydrogen, carbon, and oxygen in the a-Si deposited at the higher RF power was related to the high ion density (>10^11^ cm^−3^), which led to the removal of the hydrogen adsorbed during the growth of a-Si in addition to the removal of other impurities such as carbon/oxygen by the enhanced ion bombardment of the substrate. When the a-Si was crystallized with a UV lamp with the low-hydrogen-containing a-Si, the improved crystallinity was confirmed by Raman spectroscopy and HR-TEM, and the crystallinity of 0.8 could be obtained by Raman crystallinity.

## Data Availability

The data presented in this study are available on request from the corresponding author upon reasonable request.
